# Meltwater Pulse 1a drowned fringing reefs around Tahiti 15 000 years ago

**DOI:** 10.1098/rsos.230918

**Published:** 2023-12-13

**Authors:** Paul Blanchon, Peter Chutcharavan

**Affiliations:** ^1^ Reef Geoscience Group, Instituto de Ciencias del Mar y Limnología, Universidad Nacional Autónoma de México, Puerto Morelos, Mexico; ^2^ Department of Earth and Environmental Sciences, University of Minnesota Twin Cities, Minneapolis, USA; ^3^ Department of Geoscience, University of Wisconsin Madison, Madison, WI, USA; ^4^ Department of Geological Sciences, University of Florida, Gainesville, USA

**Keywords:** meltwater, pulse, drowned, fringing, reefs, postglacial

## Abstract

Reconstruction of postglacial sea-level rise using reef cores recovered from Tahiti during IODP Expedition 310 showed that the first major acceleration, known as Meltwater Pulse 1a (MWP-1a), was a 12–22 m rise in 340 years starting at 14.65 ka BP. Although it was reported that the pulse did not drown Tahitian reefs, the subsequent discovery of a fringing reef at the base of several cores implies that its timing, magnitude and impact require revision. Here, we report facies and paleodepth data from this reef, revise sea level, and revisit reef response. We find its reef crest is dominated by surf-adapted corals to a depth of 2.5 m and show that it retreated upslope over an approximately 1000-year interval from 16 ka. Reef development then apparently ceased at 15 ka at −106 m and remained absent for approximately 600 years, before resuming at 14.4 ka further upslope at −93 m. This absence is consistent with reef drowning and requires that MWP-1a had a smaller magnitude of 13.8 ± 1.3 m, and may have started 300 years earlier than previously reported. It confirms MWP-1a was a global event, drowning reefs on Tahiti as well as those in other oceans.

## 1. Introduction

Reconstructions of postglacial sea level (SL) are largely based on the precise dating of reef-crest corals in submerged reef sequences, following their depth adjustment for tectonic and/or glacio-isostatic overprint [1–6]. One of the first reconstructions from Barbados initially showed two rapid accelerations in SL at approximately 14 and 11 ka, which were thought to be the result of massive inputs of meltwater during ice-sheet disintegration [1]. It was later recognized that these meltwater pulses had caused the shallow reef-crest units on Barbados to drown and back-step upslope [7]. This drowning and back-stepping response has also been discovered in other areas and subsequently used constrain the timing of the meltwater pulses [7–15].

The drowning of shallow reefs is dependent upon the rate of SL rise exceeding the rate of reef-accretion for an extended period of time, until the crest is submerged below its habitat zone and enters the adjacent zone further downslope [7]. As a consequence, reef drowning is most easily recognized where the crest has a distinct and depth-restricted coralgal community, and the submergence is defined by a shift to a deeper assemblage. If this assemblage shift is accompanied by the back-stepping of reef-crest corals further upslope, then a SL jump can be reasonably inferred, and its timing, rate and magnitude estimated [7,14]. In the Caribbean, shallow wave-exposed reef crests have a distinctive zonation dominated by near-monospecific thickets of *Acropora palmata,* which extend to depths of 5 m or less, and are mixed with other corals below this [16–19]. If these ecological assemblages are perfectly preserved in the reef deposit, escaping the influence of hurricanes and other taphonomic processes, then reef drowning and SL jumps can be recognized and constrained [14].

The requirement for a distinct and depth-restricted zonation in reef-crest assemblages has posed difficulties in identifying and constraining meltwater pulses in other postglacial reef sequences. At Tahiti, cores from the barrier reef at Papeete recovered a thick reef-crest sequence of robust acroporid and pocilloporid corals intergrown with crustose coralline algae (CCA) and vermetids [2,20]. Based on modern ecological surveys, this assemblage is consistent with reef-crest zones between 0 and 6 m of SL [21]. However, in cores from IODP Expedition 310 drilled offshore to complete the postglacial reconstruction, this crest assemblage was absent due to a lack of acroporids prior to 13 ka [22]. This led to uncertainty in both SL reconstruction and assessing the response of shallow reefs, whose growth was reported to have continued uninterrupted throughout the meltwater pulse [23]. To determine the onset and magnitude of Meltwater Pulse 1a (MWP-1a) without an identifiable reef crest, Deschamps *et al*. [6] used the first coral to colonise the Pleistocene substrate in Hole M0015A, arguing for shallow-water growth (less than 5 m) based on its encrusters. They also argued that corals with ‘robust’ branching forms in Hole M0024A were coeval and had the same shallow-water encrusters. Using these coral index points, they estimated MWP-1a to be a 12 to 22 m rise with an onset of 14.65 ka BP. But given that corals can colonise the sea floor anytime after inundation, the use of basal colonies as index points introduces significant uncertainty in the accuracy of SL position during that interval [24,25].

This uncertainty in identifying the onset of MWP-1a at Tahiti has been compounded by the discovery of a fringing-reef unit at a higher elevation in cores from marginal sites [26], which were excluded from previous analyses [6,23]. This higher unit contained surf-adapted corals in direct contact with the Pleistocene substrate, providing confidence that the reef fringed the coast. Here, we report the detailed stratigraphic and facies analysis of this new fringing-reef unit using uranium-series (U-series) ages reported in a companion publication, which reconstructs the minimum SL rise across MWP-1a [27]. With this data, we reconstruct the paleoenvironment of the basal coralgal sequence at Tahiti, estimate the paleodepth of all *in-situ* corals and their reefal units, and refine the minimum SL reconstruction. We then reassess the impact of MWP-1a on fringing-reef development.

## 2. Methods

### 2.1. Tahiti offshore drill sites

**Figure 1.**
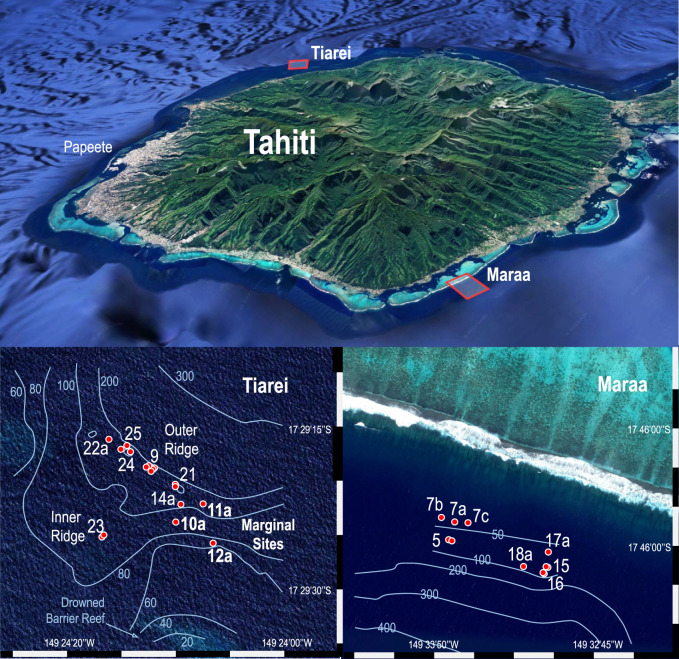
Tahiti IODP Expedition 310 drill sites. A total of 20 holes were drilled off Tiarei on the north coast and 15 off Maraa on the south coast. Note that the Tiarei Marginal Sites are downslope of a drowned section of the modern barrier reef, which initiated immediately following MWP-1a [20].

The IODP Expedition 310 to Tahiti recovered reef cores mostly from two offshore sites: Tiarei on the north coast with 20 cores and Maraa on the south coast with 15 [28]. These had a traditional recovery of approximately 58%, amounting to some 500 m of core [29]. At Tiarei, the drilling targeted two submerged ridges: a large outer ridge between the 90–110 m isobaths (Sites 9, 21 and 24–26) and a smaller inner ridge between the 65–90 m isobaths (Site 23; figure 1). Several holes were also drilled on the margin of these ridges on a featureless but sloping sea floor denominated as ‘Marginal Sites’ (Sites 10–14). On the south coast at Maraa, two transects were drilled: one in the west between the 40–80 m isobaths (Sites 5 and 7) and one in the east between the 50–100 m isobaths (Sites 15–18; figure 1). Abbey *et al*. [22] reported radiocarbon dates and coral assemblages at both Tiarei ridges and the Maraa transects, and Camoin *et al*. [23] and Deschamps *et al*. [6] reported the U-series dating and a SL reconstruction largely from the Tiarei sites. However, none of these studies included core sequences from the Tiarei Marginal Sites due to the predominance of volcaniclastic sediment and limited dating of the basal sequence. By including these Marginal Sites, Blanchon *et al*. [26] combined the existing assemblage and age data, and identified sedimentary facies, revised reef development and reported the existence of a basal fringing-reef unit.

### 2.2. Stratigraphy and facies analysis

For this study, basal sections of cores from each hole at Tahiti were re-logged and sampled in the IODP Gulf Coast Repository at TAMU in College Station, Texas, in July 2017. Core logging followed the Reef Framework Analysis Protocol outlined in Blanchon *et al.* [26], which differentiates skeletal-framework units composed predominantly of mutually supported *in-situ* corals, from detrital units composed predominantly of grain-supported clasts (following [30]). We used this protocol to identify *in-situ* corals and their framework units using combinations of presence/absence indicators including, in order of confidence, basal-attachment surfaces, coral framework fabric and consistent upward-oriented geopetals greater than 0.3 cm diameter that contained lithified sediment. We found these criteria to be reliable in massive and encrusting colonies where basal attachment surfaces are common [30], but not in branching and platy colonies where basal attachment surfaces are rare and orientation can be deceptive (especially on sloping substrates). In these cases, we found that consistency in orientation and/or mutual proximity of coral colonies was the key to a reliable differentiation of framework from detrital units [26]. By combining sedimentologic and taphonomic analysis with the taxonomy of the coralgal assemblages reported previously [22,28], we determined the sedimentary facies for each hole. Stratigraphic unit boundary elevations were also checked, and the assumed default core position at the top of the coring run was adjusted in some cases based on facies continuity and downhole imagery [29]. Elevations of previously dated samples from Deschamps *et al*. [6] in cores with adjusted positions were changed accordingly. The hiatal surface of underlying Pleistocene substrate was identified using the presence of corals with macroscopic neomorphism of mineral phases, fabric truncation due to abrasion or bioerosion, and U-series dating of well-preserved corals in the same facies. Neither elevations of stratigraphic boundaries nor dated corals were corrected for island subsidence (0.25 mm yr^−1^) given that this is minimal over the approximately 2 ka timescale considered here (following [2,6,31]).

### 2.3. Paleodepth and sea level

**Figure 2.**
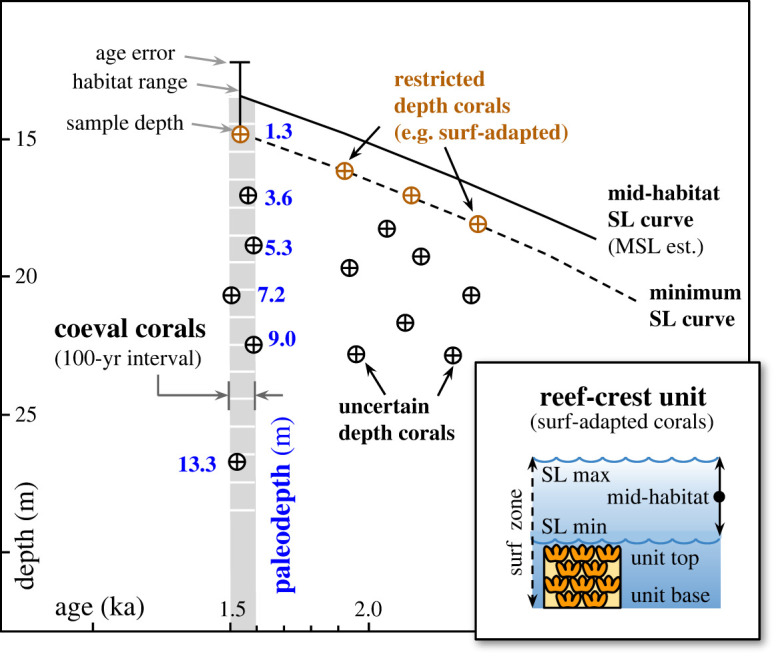
Paleodepth and sea level (SL) reconstruction from reef-crest units. Reef-crest units composed of surf-adapted corals can be used to constrain depth of the surf zone based on mean fair-weather wave height, assuming that modern wave values are representative of the past (see supplementary information). The elevation of the boundaries of the reef-crest unit can then be used as datum to reconstruct the position of SL. A ‘minimum SL’ position is represented by the top of the unit, assuming that it reached mean low water. A ‘maximum SL’ is derived from the base of the unit, assuming that it developed at the base of the surf zone. Given that neither of these assumptions may be correct, a more representative ‘mid-habitat’ SL position divides the two and can be further constrained by including an estimate of mean tidal range.

Although reconstruction of coral paleodepth in postglacial reef sequences has relied on the depth habitat of their modern counterparts [32,33], individual fossil corals are imprecise paleo SL index points because their relationship with both the SL datum and depth habitat is uncertain. Their sedimentary and paleoecological context, however, can reduce these uncertainties. Here, we reconstruct paleodepth and SL using facies units consisting of surf-adapted coral morphospecies, which allows us to delineate both a SL datum and a depth habitat. These surf-adapted morphospecies are compact-branching or reptant forms with large basal attachments, which today resist high turbulence and dislodgement in modern reef-crest and flat environments (see electronic supplementary information). We therefore consider facies units composed of these surf-adapted forms to be reef-crest units with a lower boundary at surf-base and an upper boundary not exceeding mean low water (figure 2). As such, their depth habitat can be independently derived from a calculation of surf-base using the breaking depth of fair-weather waves (see electronic supplementary information). Using this value, two paleo SL estimates can be reconstructed: first, a ‘minimum SL’ position is based on the elevation of the upper boundary of the reef-crest unit and is an estimate of the mean low water datum. Second, a ‘mid-habitat SL’ position is derived from the habitat mid-point between the upper limit of the surf-zone depth range and upper boundary of the reef unit, and is an estimate of the mean SL datum (figure 2). Given that the elevations of dated coral samples underestimate the minimum SL position, they should not be used in defining either datum, unless they occur at the upper surface of the unit. Once these estimates of paleo SL have been made, paleowater depths of all dated coral samples can be estimated by employing a coeval-coral approach (figure 2), which compares the relative depths of corals within an age window of 100 years (following [14,26]). All new U-series coral ages used here to define paleodepth and SL position are from a companion publication [27] and are provided in the supplementary information (electronic supplementary material, table S1).

## 3. Results

### 3.1. Tiarei basal-unit facies

**Figure 3.**
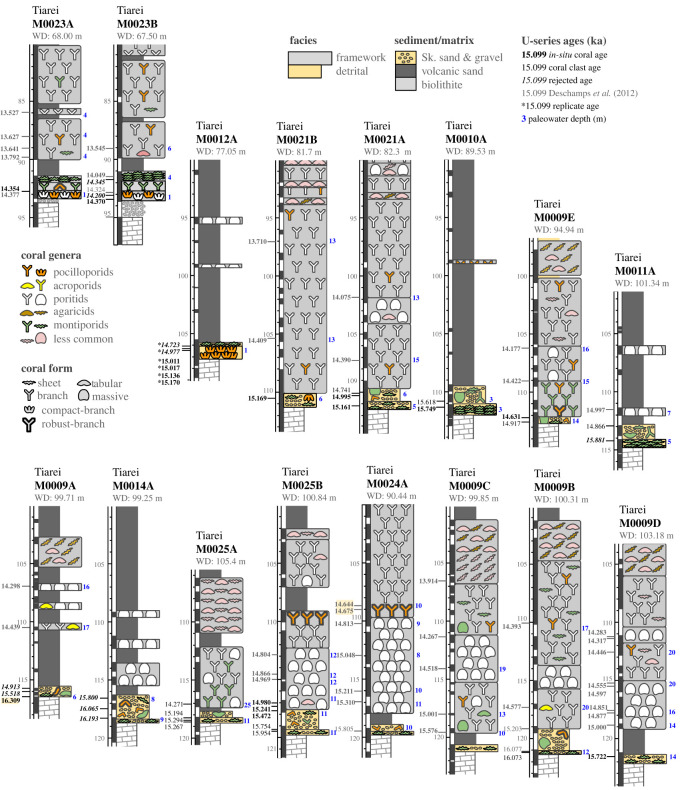
Core transects of Tiarei sites arranged by substrate depth. Core logs show sedimentary facies, coral assemblages, U-series ages (ka) and paleowater depths (in blue). Fringing-reef facies are only present at the base of the Marginal Sites 10, 11, and 12, and at the base of the Inner-Ridge Site 23. Note that the large 13.8 m and 600-year gap between the fringing reef at Site 12 and that at Site 23 is consistent with reef drowning and back-stepping during MWP-1a. Highlighted ages in Hole M0024A were used by Deschamps *et al*. [6] to identify the onset timing and magnitude of that meltwater pulse, but, as shown by our new data, they grew at paleowater depths of 10 m, which is substantially deeper than reported.

**Figure 4.**
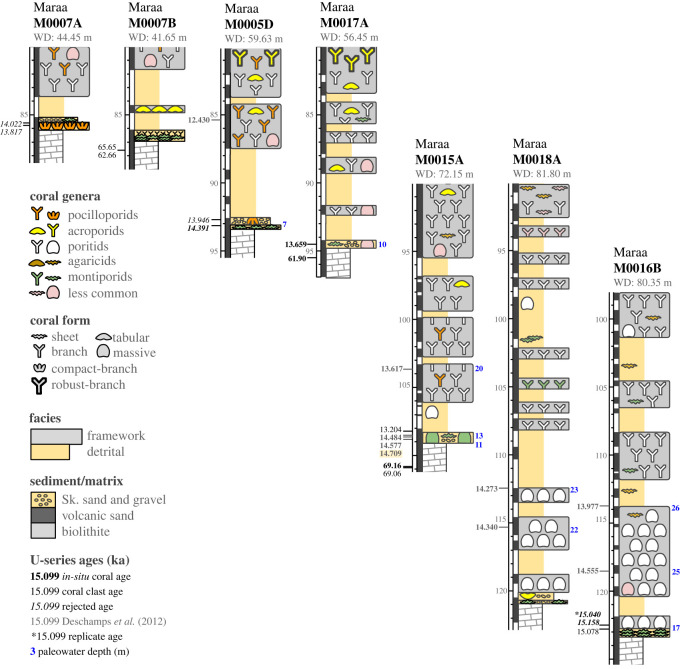
Core transects of Maraa sites arranged by substrate depth. Core logs show sedimentary facies, coral genus and growth form, U-series ages (ka), and paleowater depths (in blue). Fringing-reef facies are only present at the base of downslope Site 16, and upslope Sites 5 and 7. The highlighted age in Hole M0015A was used by Deschamps *et al*. [6] to identify the onset of MWP-1a, but, as shown by our paleowater depths, this coral grew in 10–13 m of water, which is substantially deeper than reported.

We characterized detrital and framework units at the base of 23 cores recovered from Tiarei (16 holes) and Maara (7 holes; figures 3 and 4). All sites have a 1–3 m-thick basal unit characterized by two main facies: a gravelly coralgal bindstone and a dense coralgal framestone. At Tiarei, the close core spacing shows that these basal facies form two layers in direct contact with the older Pleistocene substrate: one from depths of 122–106 m and the other from approximately 94–91 m (figure 3). At Maraa, there are fewer cores, but the two basal facies are still present in all (figure 4).

#### 3.1.1. Gravelly coragal bindstone

**Figure 5.**
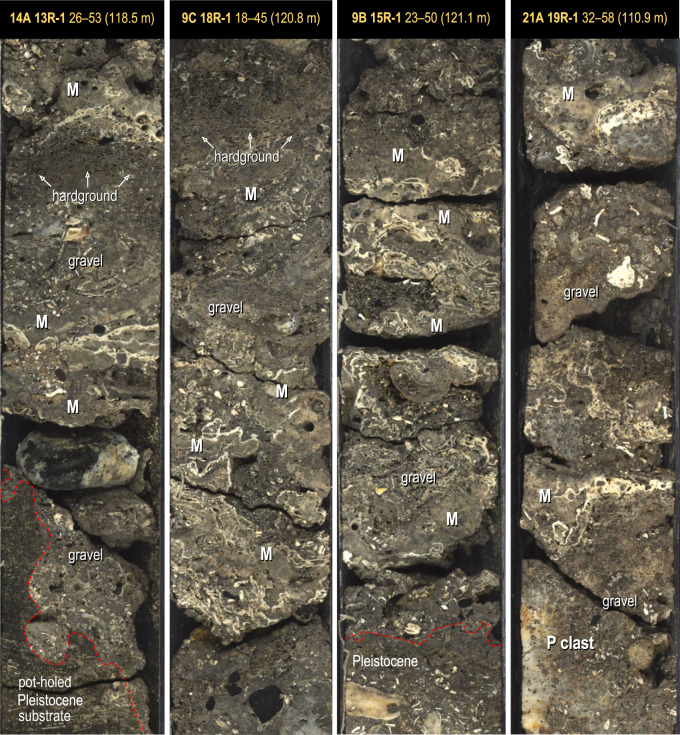
Basal unit of Tiarei cores showing the gravelly coralgal bindstone facies (*Montipora* bindstone sub-facies). This consists of thin skeletal sand-and-gravel units encrusted by *Montipora* sheets (M). Note the bioeroded hardground surface in Holes M0014A and M0009C, which signifies a depositional hiatus and/or active marine cementation.

In Tiarei cores, a predominantly detrital unit 0.5–2.5 m thick is found at all sites except for 12 and 23 (figure 3). In this unit, two sub-facies are differentiated based on the proportion of *in-situ* corals: a consolidated *Montipora* bindstone and a loose coral gravel. The *Montipora* bindstone is a 0.2–0.8 m layer at the base of the facies with a sharp and in some cases erosive contact with the underlying substrate (figure 5). It consists of a skeletal pebble-gravel colonised at closely spaced intervals (10–20 cm) by thin encrusting sheets of *Montipora* and millimetre-thin CCA. Skeletal clasts include heavily bioeroded fragments of coral, particularly pocilloporids, CCA, shells and basaltic pebbles, admixed with a skeletal-volcanic sand matrix. This sub-facies is consolidated and in some cases capped by a bioeroded hardground, signifying condensed sedimentation and active marine cementation (figure 5). Overlying it at all sites is the loose coral gravel sub-facies, which is poorly recovered. This is a 0.5–2 m-thick layer of unconsolidated pebble- to cobble-sized clasts of coral, colonised at widely spaced intervals (greater than 50 cm) by encrusting *Montipora* sheets and millimetre-thin CCA crusts. Clasts are dominated by fragments of branching and robust-branching pocilloporids, with subordinate massive favids and montastrids, and tabular and branching montiporids. All are commonly bioeroded and have millimetre-thin CCA crusts. This sub-facies has little to no matrix, likely due to washout during drilling.

#### 3.1.2. Dense coralgal framestone

**Figure 6.**
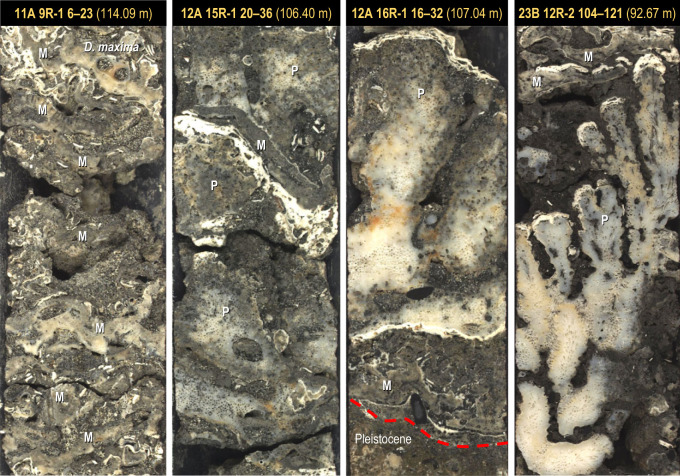
Basal unit of Tiarei cores showing the dense coralgal framestone facies. Holes M0012A and M0023B show the compact-branch *Pocillopora* framestone, and M0011A shows the encrusting *Montipora* framestone. Note that the *Pocillopora* sub-facies has an assemblage of compact-branch pocilloporids (P) such as *P. meandrina* and *P. verrucosa*, alternating with encrusting montiporids (M), showing that both colonised the surf zone. The *Montipora* framestone, by contrast, lacks pocilloporids but contains shallow vermetids such as *D. maxima*, and caps the *Pocillopora* framestone at Sites 23 and 12, implying that these sub-facies grew in both equivalent zones with higher sediment flux, as well as adjacent depth zones.

At Marginal Sites 10, 11 and 12, and the Inner-Ridge Site 23, the basal unit is a 0.7–1.0 m-thick coralgal framestone with a dense biofabric and a cemented interstitial matrix of pebbly skeletal-volcanic sand (figure 6). Two biofacies can be differentiated based on the *in-situ* coral assemblage: one containing compact-branch pocilloporids and the other encrusting montiporids. The *Pocillopora* biofacies is only found at Sites 12 and 23 and consists of small (10 cm) bioeroded colonies of *in-situ Pocillopora* spp. (*P. verrucosa, P. damicornis* and *P. meandrina*) with closely spaced, short, compact branches and large basal attachments, commonly intergrown with thin encrusting sheets of *Montipora* sp. (figure 6). Secondary encrusters include millimetre-thin CCA, sometimes intergrown with small vermetid gastropods. The *Montipora* biofacies at Sites 10 and 11 is similar but lacks pocilloporids, and consists only of thin (0.5–2 cm) wavy sheets of encrusting montiporids with millimetre-thin crusts of CCA intergrown with small vermetids, including the larger *Dendropoma maxima*.

### 3.2. Maraa basal-unit facies

#### 3.2.1. Dense coralgal framestone

**Figure 7.**
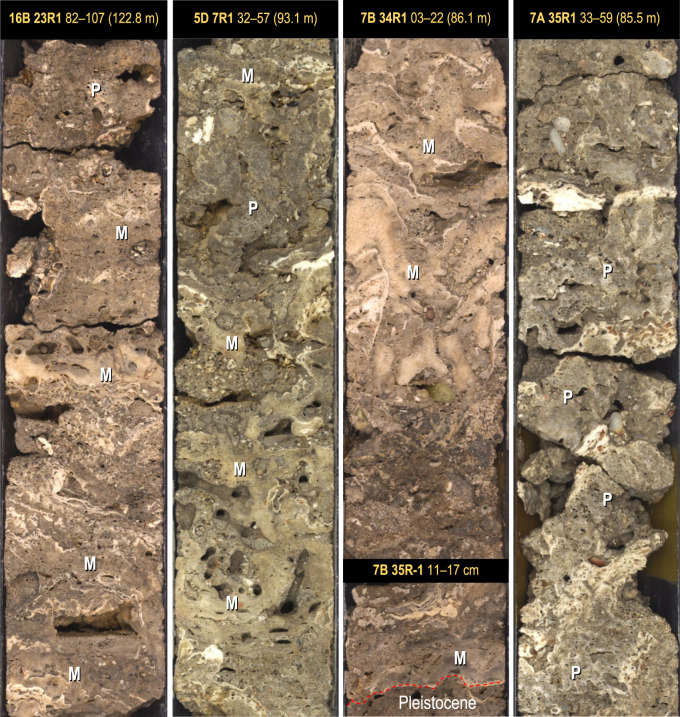
Basal unit of Maraa cores showing the dense coralgal framestone facies. The encrusting *Montipora* framestone occurs in Holes M0016B, M0005D and M0007B, and the compact-branch *Pocillopora* framestone in Hole M0007A. The separation of facies at Site 7 likely represents a fringing-reef crest at 7A (with compact-branch pocilloporids, and centimetre-thick intergrowth of CCA and vermetids) and its proximal back-reef at 7B. Note the heavy bioerosion in Holes 16B, 5D and 7A, which likely indicates a condensed sequence with low accretion rates.

On the south coast at Maraa, the facies sequence is similar, and the basal unit at Sites 7, 5 and 16 starts with a thinner (0.2–0.6 m) layer of coralgal framestone composed of either the compact-branch pocilloporid or encrusting montiporid biofacies (figure 4 and figure 7). The *Pocillopora* biofacies is only found in Hole M0007A where it forms a thin layer (25 cm) with a dense biofabric of small, highly bioeroded colonies encrusted by millimetre-thin intergrowths of CCA and vermetids in a cemented skeletal sand matrix (figure 7). In Holes M0005D, M0007B and M0016B, pocilloporids are replaced by a thin layer of the encrusting *Montipora* biofacies, also with bioeroded colonies (figure 4).

#### 3.2.2. Gravelly coralgal bindstone

Overlying the coralgal framestones at Sites 5 and 7, and forming the basal layer at Sites 15, 17 and 18 is a consolidated and thinner version (0.3–0.8 m) of the gravelly coralgal bindstone found at Tiarei. At Sites 15 and 17, this facies also contains small- to medium-sized head corals (figure 4).

### 3.3. Paleoenvironmental interpretation

**Figure 8.**
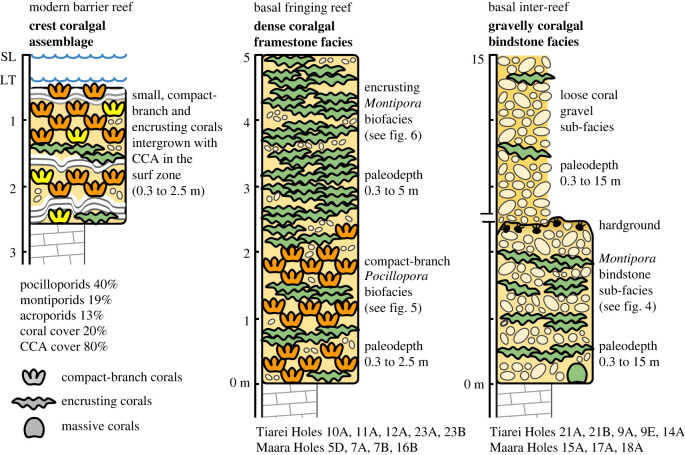
Paleoenvironment of basal facies in the Tahitian cores. Left: modern reef-crest coralgal assemblages from the barrier reef around Moorea [34] show a surf zone dominated by CCA and small, compact-branch and encrusting corals. Centre: basal fringing-reef facies in Tahitian IODP cores consist of a reef-crest unit composed of dense coralgal framestone, with characteristics similar to the modern crest assemblage, albeit lacking CCA and acroporids. Surf-adapted compact-branch pocilloporids and encrusting montiporids form a biofacies that developed to a depth of 2.5 m (surf-base) and is overlain by a biofacies composed only of encrusting montiporids. The difference between these biofacies and the modern assemblage is the paucity of CCA and acroporids, which is likely the result of increased sediment flux in a coastal fringing-reef setting [26]. Right: inter-reefal deposits are dominated by skeletal detritus bound in places by encrusting montiporids.

The dense coralgal framestone facies, with its biofabric of encrusting and compact-branch corals, is analogous to assemblages that today colonise high-energy shore-attached fringing reefs around Moorea, Tahiti's sister island (figure 8). Early ecological surveys of these reefs showed that reef-crest assemblages in 2–3 m of water are dominated by small compact-branching species of *Pocillopora* (40%) and *Acropora* (13%), with subordinate encrusting *Montipora* (19%), but that corals only have a live cover of approximately 20%, with the rest of the substrate covered by CCA [34]. Although no maximum depths were reported by these early surveys, the compact-branching morphospecies of *Pocillopora* found at Sites 7, 12 and 23 are particularly useful as environmental indicators because inter-branch spacing is related to environmental gradients in wave energy [35]. The common reef-building coral *P. damicornis,* for example, changes to a compact-branching morphospecies as it shallows into the breaker zone [35–39]. Furthermore, Sebens & Done [40] measured flow regime and coral morphology in shallow reef-crest and flat habitats, and reported that the highest flow regimes were occupied almost exclusively by small compact-branching coral morphospecies. As a consequence, the dense framework of compact-branching forms of *Pocillopora* found at Sites 7, 12 and 23 can be reliably interpreted as having developed under surf-zone conditions. The extent of this habitat can be calculated independently from the wave climate around Tahiti, where fair-weather waves of approximately 1.5 m [41] produce a surf zone in waters approximately 2.5 m deep, thereby providing a paleodepth limit for these surf-adapted forms (see electronic supplementary information).

**Figure 9.**
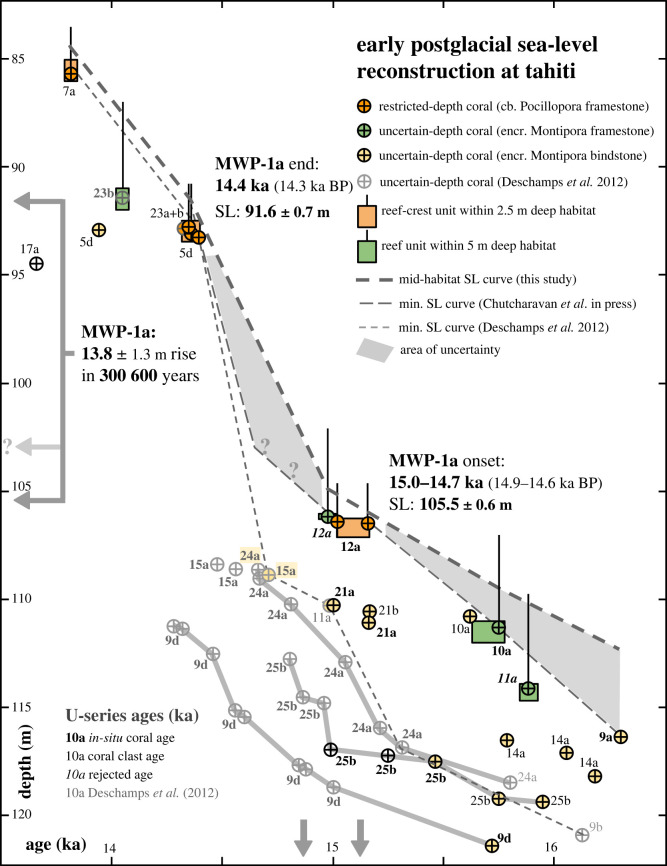
Mid-habitat SL reconstruction from Tahitian cores during the 14–16 ka interval. The elevation of fringing reef-crest units (compact-branch *Pocillopora* framestone), shown as orange boxes, are used to constrain SL position by assuming it coincides with the mid-point between the unit top (minus a 0.3 m tide level) and habitat upper limit (2.5 m; figure 9). The U-series coral ages (orange circles) constrain crest-unit age and minimum SL curve (ages in [27]; see supplementary information). The reef-crest unit in Hole 12A, and the upslope crest unit in Holes 23A,B and 5D are separated by a 12.5 m, 600-year gap in reef development, which corresponds to MWP-1a (shown by horizontal arrows). If the 12A crest unit was the last to develop, then the onset of this pulse occurred at 15 ka (14.94 ka BP); but if it retreated further upslope, then onset may be as late as 14.7 ka (14.65 ka BP), as shown by the extension of the minimum SL curve. The age and elevation of the other fringing-reef facies unit (encrusting *Montipora* framestone), shown by green boxes and circles, are used to delineate SL position either side of the meltwater pulse. Also shown are accretion trends (grey lines) for the deeper and well-dated Massive Porites unit in Holes 9D, 24A and 25B, which show a 200–300% increase around 15 ka (arrows mark inflection in accretion trends).

For the basal facies that lack surf-adapted corals, we reconstruct paleowater depth by comparing age-elevation data of *in-situ* corals in these units with the highest coeval corals in all cores (figure 9). This reconstruction shows that the encrusting *Montipora* framestone biofacies ranges in depth from 1–5 m (*n* = 5). The association of encrusting montiporids sheets with the surf-adapted pocilloporids shows that they inhabit the surf zone, but their presence as a capping layer over surf-zone corals at Sites 12 and 23 implies that this biofacies extends below surf-base to a depth of approximately 5 m. It is more difficult to interpret why compact-branch pocilloporids are absent in the encrusting *Montipora* framestone biofacies in Holes 10A and 11A at Tiarei, and 5D and 7B at Maraa. One possibility is a higher sediment flux in the surf zones at these sites, which also explains the absence of acroporids and limited development of CCA in these facies compared to the modern analogue.

The reconstruction further shows that *in-situ* corals in the gravelly coralgal bindstone facies, which forms the basal unit at all other sites, grew at depths of 0–15 m (*n* = 7). The presence of encrusting sheets of *Montipora* in this predominantly detrital facies confirms their tolerance to high sediment flux and suggests an inter-reefal habitat at or below fair-weather wave base, where the sea floor was dominated by intermittent sediment deposition (figure 8).

For the basal unit at Maraa Sites 15 and 17, which are also part of the gravelly coralgal bindstone facies, the paleowater depth reconstruction shows that their *Montipora* and *Montastrea* head corals colonised the sea floor at depths of up to 13 m, which conflicts with the 0–5 m reported previously [6].

### 3.4. Reef accretion and development

Dating of *in-situ* corals in the basal unit at all sites gives a maximum age of approximately 16.3 ka (16.24 ± 0.04 ka BP) in Hole 9A (figure 3). This is consistent with the initiation of fringing-reef facies at the Tiarei Marginal Sites 10, 11 and 12, which occurred between 15 and 16 ka. The first fringing reef to develop occurs in Hole 11A at approximately 115 m below SL, where a basal encrusting *Montipora* framestone colonised the Pleistocene substrate around 15.9 ka. The next reef upslope, also dominated by encrusting montiporids, occurs in Hole 10A at approximately 112 m around 15.8 ka. The final reef, composed of compact-branch pocilloporids, occurs in Hole 12A at 107 m around 15.2 ka. These three fringing-reef units therefore show a diachronous upslope retreat over approximately 1000 years during the gradual SL rise prior to MWP-1a (figure 3).

Accretion rates for the basal-unit facies are difficult to determine due to their limited thickness and abundance of clasts. However, multiple ages from *in-situ* corals in the fringing-reef framestone were obtained in Holes 23B and 12A (figure 3), allowing us to estimate its accretion rate. In these holes, the compact-branch *Pocillopora* biofacies has transient accretion rates of 1.2 to 7.0 mm yr^−1^, whereas the encrusting *Montipora* framestone biofacies has transient accretion rates of 1.2 to 4.3 mm yr^−1^. The highest accretion rate of 7 mm yr^−1^, however, only occurs in Hole 23B immediately following MWP-1a when sediment flux was lower. However, prior to the pulse, when the flux was higher, accretion rates for the fringing-reef facies in Hole 12A were 4.3 mm yr^−1^ or less.

**Figure 10.**
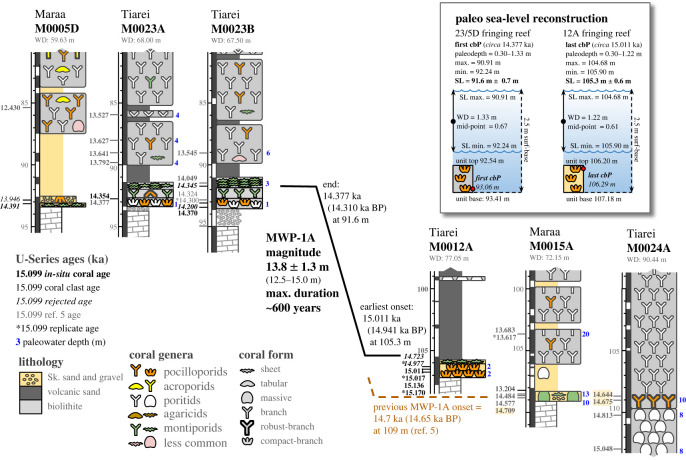
Timing and magnitude of MWP-1a. Core sequences show ages of the last-surviving coral in downslope reef-crest unit in Hole 12A, and the first colonising corals in upslope reef-crest units in Holes M0005D and M0023A/B (ages from [27]). The age-elevation difference between these crest units shows a 12.5 m, 600-year gap in fringing-reef development. Assuming SL corresponds to the mid-point of the surf-zone habitat, our paleo SL reconstruction for these units (inset) shows that MWP-1a was a 13.8 m rise in 350–600 years. Our new reef-crest data show that corals in Holes M0015A and M0024A, which were previously used to constrain timing and duration of the meltwater pulse, grew deeper than previously reported within the inter-reefal detritus.

The differences in age-elevation data between the surf-zone reef at Site 12 and the next upslope surf-zone reef at Sites 23 and 5 produce a 12.5 m and approximately 600-year gap in fringing-reef development, from 106.2 m at approximately 15.0 ka to 93.4 m at approximately 14.4 ka respectively (figure 10). This gap is consistent with the drowning and back-stepping of the fringing reef in response to the acceleration in SL rise during MWP-1a. Age data in other cores also shows a consistent response, particularly the widespread initiation and rapid accretion of the deeper Massive Porites unit, which overlies the basal unit at the deeper sites (initiation ages are approximately 15 ka; figure 3). For example, accretion-rate trends in well-dated Holes 24A, 25B and 9D show average rates of 3–4 mm yr^−1^ prior to 15 ka, but increase to 11–12 mm yr^−1^ after (figure 9).

Assuming SL corresponds to the mid-point of the habitat uncertainty of the reef-crest unit, these data show that MWP-1a was a 13.8 ± 1.3 m rise from 105.5 m to 91.6 m, starting as early as 15.0 ka (14.94 ka BP) and terminating shortly before 14.4 ka (14.31 ka BP), giving a minimum rise rate of 23 mm yr^−1^ at Tahiti (figure 10). The amount of subsidence in that time, assuming a rate of 0.25 mm yr^−1^ (Le Roy [42]), amounts to a mere 150 mm and so does not meaningfully affect the magnitude of MWP-1a [6].

## 4. Discussion

Previous analysis of the basal unit reported that a fringing-reef unit was present in all cores from Tiarei [26], but our reanalysis shows it is absent in some holes (9E, 9B, 14A and 25B), where it consists instead of the gravelly coralgal bindstone with pocilloporid clasts bound by encrusting corals. However, its presence is confirmed at all other sites where it forms a dual-zoned framework unit, with an assemblage dominated by compact-branch pocilloporids in less than 2.5 m of water and encrusting montiporids dominating from there to a depth of 5 m (figure 8). This zoned fringing reef initiated at a depth of 115 m on the steeper slopes of the Tiarei Marginal Sites at approximately 16 ka (Site 11). From there, a gradual rise in SL caused it to retreat 8–10 m upslope until 15 ka (Site 12), when shallow-reef development apparently ceased and remained absent for more than 600 years, only resuming again some 12.5 m higher at 14.4 ka (Sites 5 and 23). Absence over this interval is consistent with fringing-reef drowning due to the acceleration in SL rise at the onset of MWP-1a.

Previous assessment of SL from the Tahitian cores reported that the onset of MWP-1a occurred at 14.7 ka (14.65 ka BP), based on basal corals and their encrusters at Maraa Site 15, and mid-sequence corals at Tiarei Site 24 [6]. However, our reconstruction now shows that these corals colonised the sea floor several hundred years after substrate inundation in paleowater depths of up to 10 m. An increased paleodepth for these corals is also supported by analyses of the depth habitats of their associated encrusters, CCA and undifferentiated vermetids, which extend to depths of 10 m [22,43,44], which is double the original paleodepth estimate of less than 5 m [6]. As a consequence, these original corals and their encrusters cannot precisely constrain the magnitude or onset timing of MWP-1a.

We argue that the onset of MWP-1a occurred between 15.0 and 14.7 ka (14.94 and 14.65 ka BP). This interval represents the age difference between the youngest surf-adapted coral in the 12A fringing reef, and the ages of corals in Holes 15A and 24A used previously to constrain its onset (figure 10). The uncertainty in timing stems from two possible scenarios. The first is that the fringing reef at Site 12 was the final unit to develop, with the crest drowning at the onset of MWP-1a around 15 ka. This early-onset scenario is supported by the 200–300% increase in the accretion rate of the Massive Porites units around approximately 15 ka (figure 9), which we interpret as a response to the abrupt increase in water depth. It is also consistent with the approximately 15 ka mean and median ages of corals dated from the crest of the drowned H1d reef off Hawaii [13]. The second scenario is that the 12A fringing reef may not have been the final unit to develop and continued its upslope retreat during a slow, steady SL rise [27]. In this case, the onset of MWP-1a is more difficult to constrain and relies on the deeper corals at Sites 15 and 24, bringing it closer to the original 14.65 ka BP interpretation [6]. This is represented in figure 9 by the extension of the minimum SL curve past the 12A unit. Regardless of which of these onset scenarios is correct, it is clear that not only did MWP-1a cause a break in fringing-reef development and force it to back-step further upslope, but also it occurred approximately 4 m shallower and perhaps as much as 300 years earlier than previously reported. This means that MWP-1a not only had a smaller magnitude of 13.8 ± 1.3 m, but also may have lasted as long as 600 years, giving a rise rate of 23 to 46 mm year^−1^ [27].

The response of fringing-reef development to this meltwater pulse can be further assessed by considering the offset between its accretion rate and that of SL rise. For example, Blanchon *et al*. [26] conjectured that low accretion rates in early Tahitian fringing reefs were due to sediment suppression, and the exclusion of more sensitive but faster-growing acroporids and CCA [45,46]. This conjecture is consistent with the facies sequence in our analysis: sites with high sediment flux show no reef development, only inter-reefal skeletal detritus bound by scattered encrusting montiporids. As sediment flux is progressively reduced, the abundance of encrusting montiporids increases, until a framework is formed. Further reductions allow less sediment-tolerant species to colonize the reef, producing frameworks dominated by compact-branch pocilloporids. Taken together with the absence of *Acropora* sp. prior to approximately 14 ka, this supports a drop-out sequence in reef-crest assemblages, with acroporids and crustose corallines being the least tolerant to sedimentation, pocilloporids moderately tolerant, and montiporids most tolerant of all. This might also explain the restriction of fringing-reef development to the Tiarei Marginal Sites, where the steeper gradient of the Pleistocene substrate likely led to a reduced sediment flux compared to level substrates around the Inner- and Outer-Ridge Sites.

The suppressed accretion rate of the fringing reef also explains its upslope retreat in response to SL rise. As shown by our reconstruction in figure 9, SL rose 8–10 m between 16.3 and 15.0 ka at a rate of 6–8 mm yr^−1^, but over this interval the accretion rate of the fringing-reef unit is 4 mm yr^−1^ or less, signifying that it was forced to retreat upslope to keep pace with the rise. This retreat was facilitated by the 2.5 m-depth habitat in which surf-adapted corals survive, which provided a buffer to SL rise and gave the reef sufficient time to leave a deposit as it retreated upslope. This is because the residence time of the reef surface in the habitat range is controlled by the rate of SL rise, and so there is an inverse relation between the thickness of the fringing-reef deposit and the rate of SL rise [7]. When the rate of SL rise is high, residence time in the habitat range is insufficient for a significant thickness of reef framework to develop. At this threshold, the retreating reef disappears from the record and ‘functionally drowns’, reappearing only when the rise rate slows enough to allow a deposit to re-form. The lack of a fringing-reef deposit for 600 years between depths of 106–93 m corresponds to an increase in rise rate to at least 23 mm yr^−1^ during MWP-1a, which was apparently sufficient to prevent the fringing reef from leaving a deposit. But visiting Tahiti in the midst of the pulse would still have given a normal picture of reef development, with surf-zone corals fringing the coast. The only difference would be that drilling into those zones would show a thin intermittent layer of corals encrusting the substrate, rather than a substantive, metre-thick reef framework.

The threshold at which the rate of SL rise could induce the drowning of a reef therefore depends on both the depth habitat of the crest assemblage and its accretion rate. The best combination for constraining the timing and magnitude of a MWP-like event would be a shallow-depth habitat and/or low accretion rate, which would promote the more rapid onset of drowning. Both of these characteristics are present in the Tiarei fringing-reef unit, making it particularly well-suited for constraining MWP-1a. Conversely, for a rapidly accreting reef-crest assemblage with a larger depth habitat, like that of Tahiti's barrier-reef sequence at Papeete, a similar meltwater pulse would take longer to cause functional drowning because of the longer residence time in the deeper reef-crest habitat. Therefore, there will be an accretion-depth threshold above which the MWP will not be detected in the sequence because it was neither rapid nor large enough to submerge the reef surface into the adjacent habitat zone. This may explain the absence of MWP-1b in the Papeete barrier-reef sequence [31], especially given that a recent re-analysis of the Barbados reef sequence has shown that MWP-1b may have been both smaller in magnitude (8–11 m) and shorter in duration (250 years) than previously reported [14]. Assuming the event was similar at Tahiti, the Papeete crest assemblage, with accretion rates of approximately 16 mm yr^−1^, would have accreted 4 m in 250 years, reducing water depth over the crest to between 4 and 7 m, which is insufficient to completely submerge it below its 6 m habitat range and into a deeper assemblage. Instead, the only evidence of the pulse would be an increased accretion rate over that interval.

## 5. Conclusion

Previous assessment of the onset timing, rate and magnitude of MWP-1a from Tahiti's offshore reef sequence was not based on the identification of a distinct, depth-restricted reef-crest assemblage, but instead used deeper corals that colonised the sea floor after the meltwater pulse had started. The discovery of a fringing-reef unit with surf-adapted corals in Marginal Sites at Tiarei, and its detailed dating and sedimentary analysis presented here, now shows that MWP-1a had a smaller 13.8 ± 1.3 m magnitude, started as much as 300 years earlier (15 ka), and led to the drowning and back-stepping of this fringing reef. Even though the meltwater pulse may have lasted longer than previously thought, it still produced the same drowning response in shallow reef-crest habitats on Tahiti as it did on reefs elsewhere, like Hawaii and Barbados.

It follows therefore that distinctive, depth-restricted reef-crest units, like those reported here, are key to identifying and accurately constraining the rate and magnitude of SL rise during postglacial meltwater pulses. This stems from the restricted amount of accretion that can occur as SL rise submerges the reef-crest zone into the adjacent zone further downslope. In the optimal case, a unit of surf-adapted corals growing within 2–3 m of SL has a low accretion potential during a meltwater pulse and fails to produce a recognizable deposit, thereby undergoing functional drowning. In the less-optimal case, a more diverse reef-crest assemblage occupying a deeper habitat with a higher accretion potential cannot be fully submerged into the downslope zone by the same pulse. Sub-metre dating of these units could potentially identify pulses from increased accretion rates, but the accuracy of this approach is limited by the larger depth range of these reef-crest habitats. Therefore, it should be expected that different reef-crest assemblages with different accretion rates will produce different responses to the same meltwater pulse, with some able to maintain reef crests within their depth habitats, and others failing to do so and subsequently drowning. In the case of MWP-1a, the rate and magnitude of the pulse was clearly sufficient to cause reef drowning on a global scale.

## Data Availability

The U-series age data are provided as an electronic supplementary material file called: electronic supplementary material, table S1 U-seriesAges.xlsx [47].
